# The Rhoptry Pseudokinase ROP54 Modulates *Toxoplasma gondii* Virulence and Host GBP2 Loading

**DOI:** 10.1128/mSphere.00045-16

**Published:** 2016-03-23

**Authors:** Elliot W. Kim, Santhosh M. Nadipuram, Ashley L. Tetlow, William D. Barshop, Philip T. Liu, James A. Wohlschlegel, Peter J. Bradley

**Affiliations:** a Department of Microbiology, Immunology and Molecular Genetics, University of California, Los Angeles, Los Angeles, California, USA; b Molecular Biology Institute, University of California Los Angeles, Los Angeles, California, USA; c Department of Biological Chemistry, University of California Los Angeles, Los Angeles, California, USA; d Division of Dermatology, Department of Medicine, David Geffen School of Medicine, University of California Los Angeles, Los Angeles, California, USA; e University of California Los Angeles and Orthopaedic Hospital Department of Orthopaedic Surgery and the Orthopaedic Hospital Research Center, Los Angeles, California, USA; University at Buffalo

**Keywords:** *Toxoplasma gondii*, guanylate binding proteins, immunity-related GTPases, pseudokinase, rhoptry, virulence

## Abstract

*Toxoplasma gondii* uses unique secretory organelles called rhoptries to inject an array of effector proteins into the host cytoplasm that hijack host cell functions. We have discovered a novel rhoptry pseudokinase effector, ROP54, which is injected into the host cell upon invasion and traffics to the cytoplasmic face of the parasitophorous vacuole membrane (PVM). Disruption of *ROP54* in a type II strain of *T. gondii* does not affect growth *in vitro* but results in a 100-fold decrease in virulence *in vivo*, suggesting that ROP54 modulates some aspect of the host immune response. We show that parasites lacking ROP54 are more susceptible to macrophage-dependent clearance, further suggesting that ROP54 is involved in evasion of innate immunity. To determine how ROP54 modulates parasite virulence, we examined the loading of two known innate immune effectors, immunity-related GTPase b6 (IRGb6) and guanylate binding protein 2 (GBP2), in wild-type and ∆*rop54_II_* mutant parasites. While no difference in IRGb6 loading was seen, we observed a substantial increase in GBP2 loading on the parasitophorous vacuole (PV) of *ROP54*-disrupted parasites. These results demonstrate that ROP54 is a novel rhoptry effector protein that promotes *Toxoplasma* infections by modulating GBP2 loading onto parasite-containing vacuoles.

**IMPORTANCE** The interactions between intracellular microbes and their host cells can lead to the discovery of novel drug targets. During *Toxoplasma* infections, host cells express an array of immunity-related GTPases (IRGs) and guanylate binding proteins (GBPs) that load onto the parasite-containing vacuole to clear the parasite. To counter this mechanism, the parasite secretes effector proteins that traffic to the vacuole to disarm the immunity-related loading proteins and evade the immune response. While the interplay between host IRGs and *Toxoplasma* effector proteins is well understood, little is known about how *Toxoplasma* neutralizes the GBP response. We describe here a *T. gondii* pseudokinase effector, ROP54, that localizes to the vacuole upon invasion and is critical for parasite virulence. *Toxoplasma* vacuoles lacking ROP54 display an increased loading of the host immune factor GBP2, but not IRGb6, indicating that ROP54 plays a distinct role in immune evasion.

## Abstract (precis)

The interactions between intracellular microbes and their host cells can lead to the discovery of novel drug targets. During *Toxoplasma* infections, host cells express an array of immunity-related GTPases (IRGs) and guanylate binding proteins (GBPs) that load onto the parasite-containing vacuole to clear the parasite. To counter this mechanism, the parasite secretes effector proteins that traffic to the vacuole to disarm the immunity-related loading proteins and evade the immune response. While the interplay between host IRGs and *Toxoplasma* effector proteins is well understood, little is known about how *Toxoplasma* neutralizes the GBP response. We describe here a *T. gondii* pseudokinase effector, ROP54, that localizes to the vacuole upon invasion and is critical for parasite virulence. *Toxoplasma* vacuoles lacking ROP54 display an increased loading of the host immune factor GBP2, but not IRGb6, indicating that ROP54 plays a distinct role in immune evasion.

## INTRODUCTION

*Toxoplasma gondii* is an obligate intracellular parasite that infects approximately one-third of the human population and causes disease in immunocompromised individuals and neonates (1). *Toxoplasma* has the ability to infect a wide range of host cells and has evolved unique secretory organelles to help it to establish infection. One of these organelles is the rhoptries, which secrete proteins that form a tight junction interface between the parasite and host cell and thus mediate invasion (2, 3). In addition, the rhoptries secrete effector proteins called ROPs that are delivered into the host cytosol, which then traffic to the host nucleus or parasitophorous vacuole membrane (PVM) to coopt host signaling and innate immune pathways (4, 5). The ROP2 superfamily is the best-characterized of the ROP effector proteins and consists of more than ~40 kinases and pseudokinases, whose functions are largely unknown.

The most notable ROP kinases and pseudokinases described thus far have been shown to function in disarming the host innate immune response during infection. For example, the ROP16 kinase is injected into the host cytosol and transits to the host nucleus. ROP16 phosphorylates STAT-3 and STAT-6, which results in a decrease in production of the proinflammatory cytokine the interleukin-12–p40 (IL-12p40), thereby dampening the Th1 response against the parasite (6–8). One effector in the ROP2 superfamily whose mechanism is understood is the ROP5/17/18 complex (9–12). In contrast to ROP16, this complex of effectors traffics to the cytoplasmic face of the PVM upon injection into the host cytoplasm (10, 13). Upon reaching the PVM, they collaborate to disarm a class of cell-autonomous proteins called immunity-related GTPases (IRGs), which load onto the PVM and serve as the first line of defense against intracellular pathogens (14, 15). The IRGs are a large family of GTP-binding proteins (GBPs) that oligomerize on the PVM and cause membrane blebbing, ultimately disrupting vacuolar integrity and clearing the parasite (16). Phosphorylation of the IRGs by the ROP5/17/18 complex releases the IRGs from the PVM and protects the parasite from clearance (17). Several other ROP pseudokinases, such as ROP2 and ROP4, also associate with the PVM; however, their functions at the vacuolar membrane are unknown (18, 19). While this basic mechanism of defense against the parasite is understood, the large families of IRGs and rhoptry kinase/pseudokinases suggest that additional players are involved in a complex process of modulating cell-autonomous immunity at the PVM.

Another class of gamma interferon (IFN-γ)-dependent immunity-related loading proteins that have been shown to be important during a *Toxoplasma* infection is the GBPs (20). The GBPs have been the focus of particular interest, as the IRGs are largely absent or unlikely to play a role in human infections (e.g., there are 23 IRGs in mice but only 2 in humans, 1 of which is only expressed in testes and the other of which appears to lack GTPase activity) (21). There are 11 GBPs in mice (7 in humans), several of which have been shown to load onto the PVM during infection and are important for parasite clearance (21–23). For example, the presence of GBP1 on parasite vacuoles has been linked with membrane vesiculation and vacuole rupture (24). In addition, GBP2 has been implicated in controlling the replication of the parasites (24, 25). While type I alleles of ROP5 and ROP18 are able to diffuse GBP1 loading onto the PVM, the parasite-derived virulence factors that modulate GBP2 are unknown (22, 24).

In this report, we have identified a novel rhoptry pseudokinase, ROP54. Like other ROP effectors, ROP54 localizes to the body portion of the rhoptries and is secreted into the host cell during invasion. Upon delivery into the host cell, ROP54 traffics to the cytoplasmic face of the PVM. While disruption of *ROP54* in type I parasites shows no apparent phenotype *in vitro* and *in vivo*, *ROP54* knockouts in type II parasites grow normally *in vitro* but display a dramatic decrease in virulence *in vivo*, suggesting that ROP54 modulates some aspect of innate immunity. ROP54 does not appear to interact with the ROP5/17/18 complex and does not affect loading of IRGb6, but instead it appears to modulate the innate immune loading of GBP2 (6, 14, 26, 27). Together, the discovery and functional analyses of ROP54 provide new insight into the complex interplay between *Toxoplasma* and the interferon-inducible GTPases that regulate innate immunity.

## RESULTS

### TgME49_210370 is a novel rhoptry protein pseudokinase.

**FIG 1:**
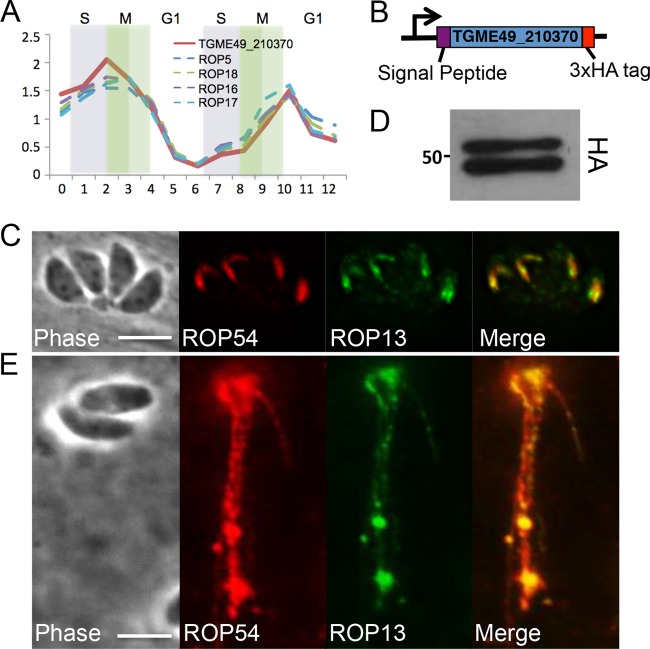
TGME49_210370 is a novel rhoptry protein. (A) The cell cycle expression profile of TGME49_210370 is similar to known *Toxoplasma* effectors. (B) Illustration of TGME49_210370 with an HA tag at its endogenous locus. (C) IFA results showing HA-tagged TGME49_210370 colocalizes with ROP13 in the rhoptries. TGME49_210370 was thus designated ROP54HA. (D) Western blot analysis demonstrated ROP54 migrates as a doublet at its predicted size (53.6 kDa). (E) Results of the evacuole assay, demonstrating that ROP54HA_II_ is secreted into the host cell, similar to the known rhoptry protein ROP13.

In examining the *T. gondii* genome for potential novel rhoptry effector proteins, we discovered a gene, designated TgME49_210370, that contained a predicted signal peptide for secretion as well as a cell cycle expression profile that was similar to known rhoptry proteins (Fig. 1A) (28). While this locus was annotated as a putative RNA helicase-1 type protein in the *T gondii* genome (or a hypothetical protein, depending on strain type), BLAST analysis did not reveal homology to any known proteins (http://www.toxodb.org) (29). We examined the amino acid sequence further by using DELTA-BLAST and Phyre-2 searches, which surprisingly indicated that TgME49_210370 was instead related to the ROP family of kinases and pseudokinases, indicating that this protein may be a more divergent member of the ROP kinase family (30, 31). The amino acid sequence for TgME49_210370 is identical between type II and III strains, with 1 amino acid change at position 112 in type I parasites. Alignment with the known rhoptry kinase ROP18 demonstrated that TgME49_210370 is missing key catalytic residues, which suggests that it functions as a ROP pseudokinase effector protein rather than a true kinase (see Fig. S1 in the supplemental material) (32).

10.1128/mSphere.00045-16.3 Figure S1 MUSCLE sequence alignment between ROP54 (amino acids [aa] 26 to 453) and ROP18 (aa 47 to 554) indicated that ROP54 is a pseudokinase. MUSCLE alignment (http://www.ebi.ac.uk/) of other known ROP kinases similarly indicated that ROP54 is a rhoptry pseudokinase (data not shown). Residues important for catalytic activity of ROP18 are underlined and shown in boldface. Download Figure S1, TIF file, 13.7 MB. Copyright © 2016 Kim et al. 2016 Kim et al. This content is distributed under the terms of the Creative Commons Attribution 4.0 International license.

To determine if TgME49_210370 is a rhoptry protein, we used endogenous gene tagging to introduce sequences encoding a 3× hemagglutinin (3×HA) epitope tag at the 3′ end of the gene of both highly virulent type I (RH*∆ku80*) and intermediate-virulence type II (Pru*∆ku80*) parasites (Fig. 1B). Evaluation in immunofluorescence assays (IFA) with anti-HA antibodies showed that TgME49_210370 localized to apical structures resembling the body portion of the rhoptries (Fig. 1C; see also Fig. S2A in the supplemental material) and colocalized with known rhoptry body proteins ROP13 and ROP7. We therefore designated TgME49_210370 rhoptry protein 54 (ROP54). Western blot analysis of ROP54HA_II_ parasites showed a reproducible doublet migrating at approximately the predicted mass of the protein lacking its signal peptide (Fig. 1D).

10.1128/mSphere.00045-16.4 Figure S2 Disruption of ROP54 in type I RH*∆Ku80* strain parasites. (A) IFA results, showing that ROP54 colocalizes with the known rhoptry protein ROP7. (B) IFA results, showing a loss in HA signal in the *∆rop54_I_* parasite clone, indicating disruption of ROP54. (C) Western blot analysis results with anti-HA antibody confirmed the loss of ROP54 in the *∆rop54_I_* clone. ROP13 was used as a loading control. Download Figure S2, TIF file, 10 MB. Copyright © 2016 Kim et al. 2016 Kim et al. This content is distributed under the terms of the Creative Commons Attribution 4.0 International license.

For ROP54 to be a potential effector protein, it must be secreted into the host cell, as typically seen with other ROP effectors (5, 33). To evaluate whether ROP54 is an injected effector, we carried out “evacuole” assays, in which parasites are unable to invade due to inhibition by cytochalasin D (CytoD) treatment but still able to release streams of rhoptry proteins into the cytosol of the host cell (10, 33). Using ROP54HA_II_ parasites, we were able to observe classic “strings” of HA-positive evacuoles emanating from CytoD-arrested parasites (Fig. 1E). These evacuoles were also positive for ROP13, which is known to be secreted into the host cell in evacuoles (33). Similar results were obtained when an evacuole assay was performed with ROP54HA_I_ parasites (data not shown). Thus, we conclude that ROP54 is injected from the rhoptry body into the host cell.

### ROP54 associates with the PVM after being injected into the host cell.

**FIG 2:**
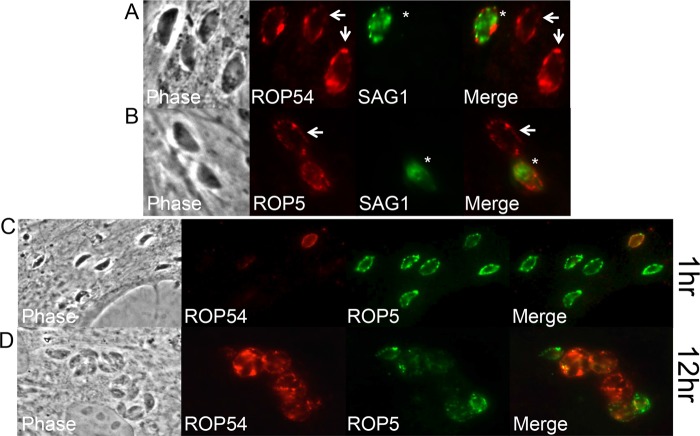
Selective permeabilization demonstrated that ROP54 localizes to the PVM. (A) Digitonin permeabilization of HFFs infected with ROP54HA_II_ parasites for 12 h showed that ROP54 is present on the cytoplasmic face of the PVM (arrow). Overpermeabilized vacuoles were SAG1 positive and are annotated with an asterisk. (B) ROP5 control for vacuolar membrane localization under digitonin treatment conditions. (C and D) HFF monolayers were infected with ROP54HA_II_ parasites and then fixed and selectively permeabilized with digitonin 1 h postinfection (C) or 12 h postinfection (D). Whereas ROP5 localized relatively early on the PVM, ROP54 was more frequently found at later time points.

Once they reach the host cytoplasm, rhoptry effectors are known to target specific intracellular compartments, including the cytoplasm, nucleus, or the PVM (6, 10, 13, 26, 33). As some of the best-studied rhoptry kinases and pseudokinases traffic to the PVM and anchor to it using amphipathic α-helices in the N-terminal region of the proteins, we examined the ROP54 sequence for putative α-helices that could mediate PV association (13). We identified two such regions, from residues 83 to 120 and 123 to 155 (see Fig. S3A in the supplemental material) that might form amphipathic α-helices when plotted on a helical wheel predictor (see Fig. S3B). To assess whether ROP54 traffics to the cytoplasmic face of the vacuolar membrane, similar to other rhoptry effectors (i.e., ROPs 2/4/5/7/17/18), we examined ROP54HA_II_ in early invasion and digitonin semipermeabilization assays (Fig. 2A) (10, 26, 34). Digitonin treatment is able to selectively permeabilize the host plasma membrane but not the vacuolar membrane or parasite membranes, enabling detection of the vacuolar membrane effectors that face the host cytoplasm. As controls, we similarly examined the rhoptry pseudokinase ROP5, which is known to traffic to the PVM, and we also utilized staining for the parasite surface antigen SAG1 to show that the vacuoles being evaluated were not breached by digitonin treatment, as the degree of permeabilization varied within individual cells on the coverslip in these experiments (Fig. 2A and B) (13).

10.1128/mSphere.00045-16.5 Figure S3 ROP54 has arginine-rich regions that resemble RAH domains. (A) Predicted amino acid sequence of ROP54 (yellow, signal peptide; white, arginine-rich domains). (B) Helical wheel prediction of underlined regions (amino acids 83 to 120 and 123 to 155) of the ROP54 amino acid sequence (http://rzlab.ucr.edu/scripts/). Download Figure S3, TIF file, 8.2 MB. Copyright © 2016 Kim et al. 2016 Kim et al. This content is distributed under the terms of the Creative Commons Attribution 4.0 International license.

Using these assays, we were able to demonstrate that ROP54 traffics to the cytoplasmic face of the PVM (Fig. 2A and B). We also observed that ROP54 is less frequently detected on the PVM relative to ROP5 at 1 h postinfection (Fig. 2C). The differences seen between the effectors may be due to fewer vacuoles being targeted by ROP54 than ROP5, although we cannot exclude the possibility that these differences are merely due to levels of detection, since ROP5 is encoded in a multicopy gene and *ROP54* appears to be present in a single copy and is likely expressed at lower levels. However, at 12 h postinfection, ROP54 can be detected on the PVM, similar to ROP5 (Fig. 2D). This suggests that ROP54 may load onto the PVM later than that seen for ROP5, perhaps requiring another partner to traffic to the PVM.

To further examine trafficking of ROP54 to the PVM, we exogenously expressed the protein in human cells with an HA epitope tag and assessed its localization to the PVM following *T. gondii* infection (see Fig. S4A in the supplemental material). Whereas ROP5 is targeted to the PVM under these conditions (33), ROP54 remained diffuse in the cytoplasm and was not detected in significant amounts on the PVM (see Fig. S4B). Because we could not be certain of the precise N terminus of ROP54 following cleavage of its signal peptide and any potential prodomains, we constructed two deletions that might expose the charged regions present in the N terminus of the protein (Fig. S4C and D), but these truncated proteins also failed to traffic to the PVM (data not shown).

10.1128/mSphere.00045-16.6 Figure S4 Exogenously expressed ROP54 does not localize to the PVM upon infection. (A) Gene model showing ROP54 lacking its predicted signal peptide (amino acids [aa] 1 to 26). This construct was transiently transfected into HT1080 cells. (B) IFA results demonstrating cytoplasmic localization of ROP54 (aa 27 to 479) in HT1080 cells upon infection with *∆rop54_II_* parasites. (C) Two N-terminally truncated ROP54 constructs were also used for ectopic expression in HT1080 cells. (D) Western blot results with host cell lysate with ectopically expressed truncated constructs of ROP54 from experiments shown in panels A and C. Download Figure S4, TIF file, 6.5 MB. Copyright © 2016 Kim et al. 2016 Kim et al. This content is distributed under the terms of the Creative Commons Attribution 4.0 International license.

### ROP54SF_II_ immunoprecipitation suggests it functions independently from the ROP5/17/18 complex.

**FIG 3:**
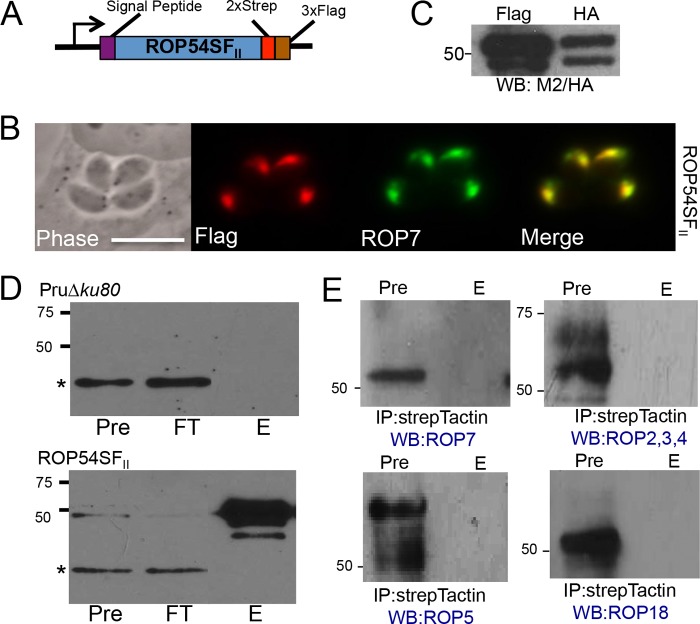
Purification of ROP54 indicated that there is no robust interaction with other known ROP effector proteins. (A) Illustration showing the endogenously tagged ROP54 with predicted signal peptide, coding region, and C-terminal 2×Strep 3×Flag epitope tags. (B) IFA with anti-Flag antibody showed colocalization with the rhoptry protein ROP7. (C) Western blot assay results for ROP54SF_II_ and ROP54HA_II_ parasite lines demonstrated that the slower-migrating band was the main band of ROP54. (D) Western blotting results with precolumn (Pre), flowthrough (FT), and elution (E) fractions of the Pru*∆ku80* (top) and ROP54SF (bottom) StrepTactin pulldown product probed with mouse anti-Flag antibody. A nonspecific band is represented by the asterisk. (E) IP-Western blot probing for known ROP kinases and pseudokinase after ROP54SF pulldown.

To identify the binding partners of ROP54, we engineered an endogenous tagging construct that would add sequences encoding a 2×Strep 3×Flag epitope tag at the C-terminal end of the ROP54 gene (Fig. 3A). The tagged ROP54 properly localized to the rhoptry body, and therefore the strain was designated ROP54SF_II_ (Fig. 3B). We additionally analyzed ROP54SF_II_ by Western blotting, which revealed a doublet that was enriched for the slower-migrating band (Fig. 3C), suggesting that this is the primary product of ROP54. To determine if ROP54 interacted with the ROP5/17/18 complex or other members of the ROP kinase family, we purified ROP54 by using a Strep-Tactin column and eluted the ROP54 complex with desthiobiotin (10). Western blot analysis of the precolumn (pre) and elution (E) fractions with an anti-Flag antibody demonstrated a significant enrichment of ROP54 relative to the untagged control (Fig. 3D). The fractions were evaluated for known ROP kinases or pseudokinases (ROPs 5/18 as well ROPs 2/3/4 and ROP7), and none was enriched in our immunoprecipitation (IP)-Western blotting or mass spectrometry data (Fig. 3E; see Table S2 in the supplemental material). These results suggest that ROP54 functions independently of the ROP5/17/18 complex and ROPs 2/4/7 on the PVM, although we cannot exclude more transient interactions that would have been disrupted during isolation. Mass spectrometric analysis of the ROP54 pulldown product did not identify any other known active kinases that may work in conjunction with ROP54. We did identify the small amounts of the inactive kinase ROP24 as well as another hypothetical protein with a predicted signal peptide (TGME49_237180), but tagging of these proteins suggested dense granule localization, and thus they were not pursued further (data not shown).

### Disruption of ROP54 in type I parasites does not affect growth *in vitro* or virulence *in vivo.*

To determine the function of ROP54, we disrupted its gene in ROP54HA_I_ parasites by homologous recombination. To do this, we utilized a knockout construct consisting of the ROP54 flanking regions surrounding the selectable marker dihydrofolate reductase (DHFR). The linearized construct was transfected into ROP54HA_I_ parasites, and knockouts were screened for loss of the HA tag. Parasite clones that lacked HA staining were isolated and verified by IFA and Western blot analysis (the resulting strain was designated ∆*rop54*_I_ [see Fig. S2B and C in the supplemental material]). No gross defects were observed in parasite intracellular growth, as evaluated in plaque assays over a 6-day period of the lytic cycle (data not shown). To determine if this disruption affected virulence *in vivo*, a small number of the ∆*rop54*_I_ parasites (~10 parasites) was injected into mice, and all of the mice died at 11 days postinfection, similar to that seen with control parasites (data not shown). Thus, loss of ROP54 does not appear to impact growth or virulence in type I parasites.

### ROP54 is not required for normal *in vitro* growth of type II parasites.

**FIG 4:**
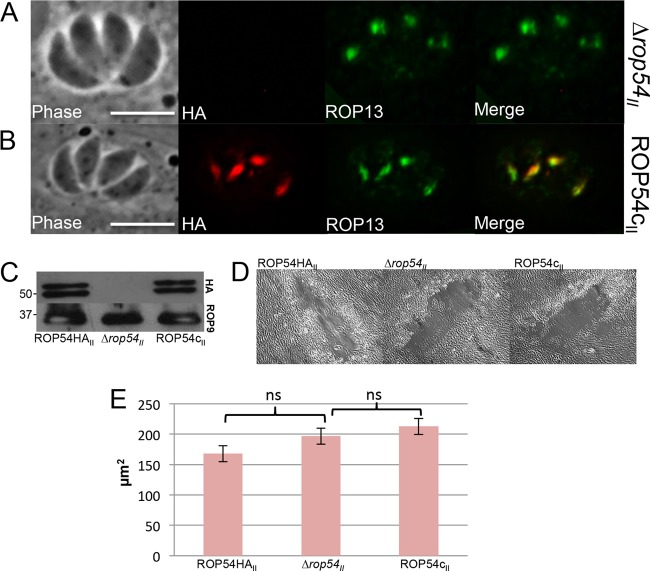
Disruption of *ROP54* in type II parasites does not affect growth *in vitro*. (A) IFA results, demonstrating the loss of ROP54HA_II_ staining in a ∆*rop54*_II_ clone. (B) IFA results for ∆*rop54*_II_ parasites complemented with *ROP54HA_II_* at the *ku80* locus (designated ROP54c_II_). Proper localization of ROP54 in the ROP54c_II_ parasite clone was assessed by colocalization with ROP13. (C) Western blot assay results, demonstrating loss of HA signal in ∆*rop54*_II_ parasites and restoration of HA signal for ROP54c_II_ parasites. ROP9 is shown as a loading control. (D and E) HFF monolayers were infected with ROP54HA_II_, ∆*rop54*, or ROP54c_II_ parasites, and plaques were visualized after 10 days. All strains exhibited similar overall fitness *in vitro* (representative plaques are shown in panel D). The area of 30 plaques from each parasite line was measured, and no significant difference (*P* > 0.05) was determined by one-way ANOVA. ns, not significant (E).

The hypervirulence of type I parasites is largely due to the robust activity of the ROP5/17/18 complex, which inactivates IRGs that would otherwise load onto the PVM, disrupt the vacuolar membrane, and clear the parasite (10, 26). Since the effects of type I ROPs 5/17/18 may mask the importance of ROP54 in parasite virulence, we assessed the function of ROP54 as an intermediate virulence type II strain (10, 14, 26). To do this, we disrupted ROP54 in Pru∆*ku80* parasites and confirmed the knockout by IFA and Western blotting (Fig. 4A and C). A *ROP54-*complemented strain (ROP54c_II_) was generated by expressing *ROP54HA_II_* driven from its endogenous promoter (Fig. 4B). The complementation construct was observed to target the *Ku80* locus, thereby excluding potential polar effects in the ∆*rop54*_II_ strain. A clonal isolate of ROP54c_II_ was evaluated by IFA, and it showed apical staining of the 3×HA epitope tag that colocalized with ROP13. The strain was also assessed by Western blot analysis, which demonstrated expression levels nearly identical to those of the parental ROP54HA_II_ parasites (Fig. 4C). To examine the role of ROP54 in *in vitro* growth, the ROP54HA_II_, ∆*rop54*_II_, and ROP54c_II_ lines of parasites were evaluated by plaque assay, and no apparent differences in growth rate were detected between the three strains (Fig. 4D and E).

### Disruption of ROP54 in type II parasites dramatically decreases virulence *in vivo*.

**FIG 5:**
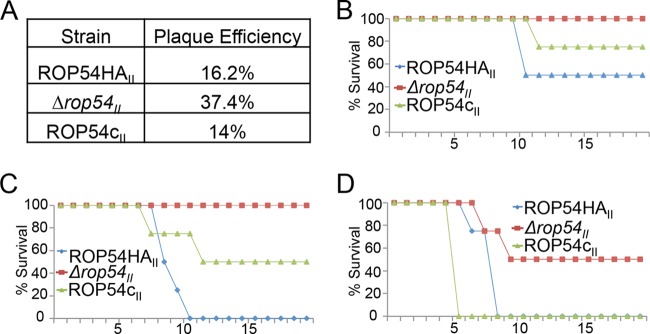
Disruption of ROP54 results in a dramatic decrease in virulence *in vivo*. (A) A plaque assay was used to verify viability of parasites injected into mice. More viable ∆*rop54_II_* parasites were injected into the mice than into the controls. A total of 500 (A), 5,000 (B), or 50,000 (C) ROP54HA_II_, ∆*rop54_II_*, or ROP54c_II_ parasites were i.p. injected into C57BL/6 mice. An ~100-fold decrease in virulence was observed between ROP54HA_II_ (50% lethal dose [LD_50_] of 500 parasites) and ∆*rop54_II_* (LD_50_ of 50,000 parasites). Virulence was mostly restored with complementation of ROP54.

To evaluate the effect of the knockout *in vivo*, mice were infected with doses of 500, 5,000, and 50,000 parasites of the ROP54HA_II_, ∆*rop54*_II_, or ROP54c_II_ strain. To ensure that any attenuation of virulence was not due to viability of the knockout or counting errors, plaque assays were performed on the parasites used for the infections, which demonstrated comparable amounts of parental and complemented strains but ~2-fold higher numbers of plaques with the knockout, demonstrating that even more knockout parasites were injected than wild-type or complemented strain parasites (Fig. 5A). Interestingly, *∆rop54_II_* parasites exhibited a 2-log reduction in virulence compared to the parental line (Fig. 5B to D). This defect was mostly restored in the complemented strain, showing that ROP54 plays an important role in virulence *in vivo* in type II strain parasites. Finally, we evaluated whether ∆*rop54*_II_-infected mice were protected against a lethal challenge with 10,000 RH*∆ku80* parasites, and all mice survived the challenge (data not shown).

### ∆*rop54_II_* parasites are more susceptible to innate immune clearance.

**FIG 6:**
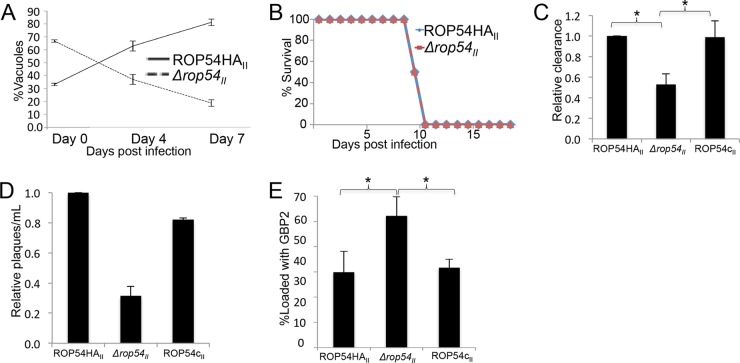
ROP54 modulates IFN-γ-dependent parasite clearance through the interference of GBP2 loading on the PV. (A) *In vivo* competition assay results for ROP54HA_II_ and *Δrop54_II_* parasite lines, showing a steady increase in the percentage of ROP54HA_II_ vacuoles and a steady decrease in the percentage of *Δrop54_II_* vacuoles as the coinfection progressed (*n* = 6, from two independent experiments). (B) IFN-γR^−/−^ mice were injected with 5,000 parasites of ROP54HA_II_ or ∆*rop54_II_* and became moribund with the same kinetics, suggesting that ROP54 modulates an IFN-γ-dependent response (*n* = 4). (C) RAW 267.4 cells were activated with IFN-γ and LPS for 24 h. The parasite strains ROP54HA_II_, ∆*rop54*_II_, and ROP54c_II_ were used to infect the cells for 20 h at an MOI of 1. qPCR demonstrated an ~50% decrease of ∆*rop54_II_* parasites relative to levels with the parental and complemented strains. Significance was determined by a one-way ANOVA. *, *P* < 0.05 (*n* = 3). (D) Primary BMDMs were activated with IFN-γ and LPS for 24 h. The strains ROP54HA_II_, ∆*rop54_II_*, and ROP54c_II_ were used to infect the cells for 20 h at an MOI of 1. Parasites were liberated by manual disruption and quantitated in a plaque assay. Values were normalized to ROP54HA_II_, and a decrease in *∆rop54_II_* viability was demonstrated (*n* = 2). (E) MEFs were primed with IFN-γ and LPS. The ROP54HA_II_, ∆*rop54_II_*, and ROP54c_II_ parasite lines were used to infect the cells for 12 h. The proportion of GBP2 loading on the vacuoles of ∆*rop54_II_*-infected cells was significantly increased, based on a one-way ANOVA. *, *P* < 0.05 (*n* = 3). The decrease in loading was restored to wild-type levels upon complementation.

To determine the kinetics of *∆rop54_II_* clearance *in vivo*, we performed an *in vivo* competition assay. We intraperitoneally (i.p.) injected a mixture of ROP54HA_II_ and strain *∆rop54_II_* parasites into C57BL/6 mice at a dose of 50,000 parasites per mouse (~40/60 ratio of ROP54HA_II_/∆*rop54_II_*). At days 4 and 7 postinfection, we euthanized mice and performed a peritoneal lavage to collect the parasites from the peritoneum and assess the ratio of ROP54HA_II_ to *∆rop54_II_* parasites by IFA. The *∆rop54_II_* parasites were outcompeted by the ROP54HA_II_ parasites *in vivo* as the infection progressed (Fig. 6A). In parallel to peritoneal lavage, spleens were harvested from animals euthanized on day 7, and ROP54HA_II_ versus *∆rop54_II_* parasite burdens were quantitated by IFA; the results showed similar parasite vacuole ratios to those found in the peritoneal lavage experiment (see Fig. S5 in the supplemental material). The decrease in relative amounts of *∆rop54_II_* parasites suggests that *∆rop54_II_* parasites either grow poorly *in vivo* or are cleared by the innate immune response.

10.1128/mSphere.00045-16.7 Figure S5 *In vivo* competition of ROP54HA_II_ and *Δrop54_II_* showed similar ratios of parasites in the peritoneum versus spleen. Day 7 spleens from the *in vivo* competition assay were harvested to evaluate relative amounts ROP54HA_II_ and *Δrop54_II_* parasites. No significant difference was observed when comparing the relative amounts of ROP54HA_II_ and *Δrop54_II_* parasites in the peritoneal lavage fluid and spleen at day 7. Conditions were evaluated using a one-way ANOVA (*P* > 0.05). Download Figure S5, TIF file, 2.2 MB. Copyright © 2016 Kim et al. 2016 Kim et al. This content is distributed under the terms of the Creative Commons Attribution 4.0 International license.

To resolve these two possibilities, we examined the virulence of ROP54HA_II_ and *∆rop54_II_* parasites in IFN-γ receptor-deficient (IFN-γR^−/−^) mice. We predicted that the virulence of *∆rop54_II_* parasites would mimic that of the parental line if virulence were dependent on an IFN-γ-mediated immune response (but would still be dramatically lower if merely due to a reduction in growth *in vivo*). To test this, we i.p. injected 5,000 ROP54HA_II_ or *∆rop54_II_* parasites separately in IFN-γR^−/−^ mice and observed their morbidity. The IFN-γR^−/−^ mice demonstrated identical morbidity kinetics when infected with either ROP54HA_II_ or *∆rop54_II_* parasites (Fig. 6B). These data demonstrated that IFN-γ signaling is necessary for the difference in virulence of ROP54HA_II_ and *∆rop54_II_* parasites and suggest that ROP54 enables parasites to evade an IFN-γ-mediated immune response (14).

To determine whether *∆rop54_II_* parasites are deficient in the avoidance of the host innate immune response, we examined ROP54HA_II_, *∆rop54_II_*, and ROP54c_II_ parasites in primed macrophages, which are the primary immune cell type infected *in vivo* (14, 35). To assess macrophage-mediated clearance *in vitro*, we infected activated murine macrophages with ROP54HA_II_, *∆rop54_II_*, and ROP54c_II_ parasites, isolated genomic DNA, and calculated the relative amount of parasite genomic DNA via quantitative PCR (qPCR) at 20 h postinfection. We observed a 2-fold decrease in the relative amount of *∆rop54_II_* genomic DNA compared to the ROP54HA_II_ and ROP54c_II_ parasite lines (Fig. 6C) (36–38). To determine if the decrease in *∆rop54_II_* genomic DNA correlated with a decrease in *∆rop54_II_* parasite viability, we similarly assessed the viability of ROP54HA_II_, *∆rop54_II_*, and ROP54c_II_ parasites within activated macrophages under the same conditions. We mechanically disrupted the macrophages to liberate the parasites from the cells and measured parasite viability in plaque assays (38). In agreement with the PCR results, we observed a substantial decrease in the *∆rop54_II_* parasite viability relative to the controls (Fig. 6D), indicating that ROP54 enhances the ability of the parasite to avoid macrophage clearance.

### The loss of virulence in ∆*rop54_II_* parasites correlates with GBP2 loading.

Since ROP54 localizes to the PVM upon invasion (Fig. 2A) and aids in the avoidance of an innate immune response, we investigated whether ROP54 potentially interfered with the function of IRGs (10, 14, 27, 39). We first wanted to determine if IRGb6 and ROP54 were both present on the PVM during the course of a *Toxoplasma* infection. To test this, ROP54HA_II_ parasites were used to infect activated macrophages for 1 h and 12 h. The cells were assessed by IFA, and colocalization of ROP54 and IRGb6 was observed at both time points (see Fig. S6A in the supplemental material). To determine whether ROP54 disrupted IRGb6 loading, we quantified the loading events between ROP54HA_II_ and *∆rop54_II_* parasites in activated macrophages (14). However, no difference was observed with the loading of IRGb6 between ROP54HA_II_ and *∆rop54_II_* parasites (see Fig. S5B in the supplemental material).

10.1128/mSphere.00045-16.8 Figure S6 ROP54 does not modulate loading of IRGb6 onto PVM. (A) Colocalization of ROP54 and IRGb6 (1 h and 12 h postinfection) was demonstrated in macrophages activated with IFN-γ and LPS. (B) IRGb6-positive vacuoles were enumerated and compared between ROP54HA_II_ and *∆rop54_II_* parasites. No difference in loading was observed. Download Figure S6, TIF file, 6.9 MB. Copyright © 2016 Kim et al. 2016 Kim et al. This content is distributed under the terms of the Creative Commons Attribution 4.0 International license.

We also investigated a different family of immune loading proteins called p65 GBPs. To determine if ROP54 enables parasites to evade the antimicrobial effects of GBP2, we compared the immune loading of GBP2 on ROP54HA_II_, *∆rop54_II_*, and ROP54c_II_ parasites. We predicted that if ROP54 modulated GBP2 loading, we would observe a difference in loading between the *∆rop54_II_* parasites and the controls. To examine loading of GBP2, we activated mouse embryonic fibroblasts (MEFs) and infected the cells with ROP54HA_II_, *∆rop54_II_*, or ROP54c_II_ parasites. IFA analysis with anti-GBP2 antibodies showed a substantial increase in the percentage of *∆rop54_II_* vacuoles loaded with GBP2 compared to that in the ROP54HA_II_, and ROP54c_II_ vacuoles (Fig. 6E). These data indicate that ROP54 is a virulence factor that plays a role in evading the cell-autonomous immune mechanism of GBP2.

## DISCUSSION

The family of *Toxoplasma* ROP kinases and pseudokinases has largely been identified by traditional organelle isolation and antibody production strategies, as well as more recent proteomic and bioinformatics approaches (4, 40, 41). Together, these studies have determined that the ROP2 superfamily consists of more than 40 rhoptry kinases and pseudokinases (41). While the functions of most of these proteins are unknown, analyses of just a few of these family members have shown that they are key players in *T. gondii*’s ability to hijack host functions and evade innate immunity (9). In this work, we identified ROP54 by screening the *T. gondii* genome to find potential rhoptry proteins based on the criteria of the presence of a predicted signal peptide and a cell cycle expression profile similar to that of other known ROPs (9, 42). ROP54 appears to be a member of the ROP kinase family, as it contains a predicted ROP2-like kinase fold, based on DELTA-BLAST and Phyre-2 analyses, and it is most likely a pseudokinase, as it lacks the key amino acids of the kinase catalytic pocket (see Fig. S1 in the supplemental material) (30, 31). We were unable to find other divergent ROP kinase family members using this approach or by BLAST searches with ROP54, but it is possible that other proteins have diverged even further and were thus unrecognized by these searches.

We verified rhoptry localization for ROP54 by C-terminal endogenous gene tagging, and the results were consistent with those for other ROP kinases that are generally amenable to epitope tagging at this terminus (Fig. 1C and 3B; see also Fig. S2A in the supplemental material). The tagged protein migrates as a doublet on Western blots, although this doublet was diminished in the 2×Strep 3×Flag-tagged protein (Fig. 3C). The doublet is not likely due to processing of a prodomain, as seen with other ROPs, as there are no predicted processing sites that are apparent in the N-terminal region of the protein that could give rise to the observed banding pattern (43, 44). In addition, the ratio of the two bands was not consistent with the pattern seen for other rhoptry prodomain processing events (32, 33).

We were able to show that ROP54 is injected into the host cytosol in a evacuole assay, indicating that it is a rhoptry effector protein (as opposed to a resident rhoptry protein that is not secreted) (Fig. 1E). Upon injection into the host cytoplasm, ROP54 appears to associate with the vacuolar membrane (Fig. 2A). Interestingly, ROP54 staining is observed on fewer vacuoles than ROP5 at early time points in invasion (~1 h), but ROP54 staining is more prevalent at later time points (12 h) (Fig. 2C and D). We were unable to accurately quantitate these differences in ROP5 and ROP54 staining at early time points due to the difficulties in detection of low levels of ROP54 on the PVM in these experiments. One possible reason for these differences is that ROP5 is highly expressed with 9 to 10 tandem copies of the gene in type II parasites and thus is more readily detected than a single copy of ROP54 (12). ROP5 is also likely present at a high frequency on the PVM at early time points, because it protects the parasite from the early loading IRGs and clearance (12, 17). The better detection of ROP54 at later time points may also be due to cooperative loading with parasite or host binding partners (e.g., other ROPs, GBPs, or IRGs) that may be important for ROP54 function or may simply reflect detection of the protein.

In spite of having arginine-rich regions in the N-terminal portion of the protein that might function similar to RAH (arginine-rich amphipathic helix) domains (see Fig. S3 in the supplemental material), exogenously expressed ROP54 appears to remain cytosolic and does not traffic to the PVM upon infection (see Fig. S4 in the supplemental material) (26). As we could not exclude processing events that would result in correct positioning of the arginine-rich region, we tested various N-terminal truncations, but these also did not result in vacuolar targeting. It is still formally possible that a precise N terminus is required for ROP54 vacuolar association, although other ROP RAH domains appear to be much more robust and tolerate N-terminal fusions as well as deletions of subregions of the key trafficking helices (13). Alternatively, association of ROP54 with the vacuolar membrane may require other parasite- or host-derived partners.

To address whether ROP54 acts by interacting with other ROP kinases, we immunoprecipitated the protein using ROP54SF_II_ strain parasites (Fig. 3). While we anticipated that we might immunoprecipitate an active rhoptry kinase, we did not find detectable amounts of the ROP 5/17/18 complex or other known active ROP kinases. This is in agreement with tandem affinity purification pulldown products of ROP 5/17/18, which also do not coprecipitate with ROP54 (10, 14). We did immunoprecipitate low amounts of ROP24 and TGME49_237180, although the significance of these partners is unclear, as they appear to have localizations reminiscent of GRA proteins based on epitope gene tagging (data not shown). The localization of these proteins should be taken with some caution, however, as ROP24 and TGME49_237180 have cell cycle expression profiles similar to ROPs, which suggests that the epitope tags are mislocalizing the proteins (28, 45). It is also possible that the interactions of ROP54 and its bona fide partners are transient or weaker than those of the ROP5/17/18 complex and its host substrates. Ultimately, identification of the interactions between ROP54 and its parasite and host partners will best reveal how it functions in *Toxoplasma*.

Disruption of *ROP54* in highly virulent type I parasites leads to no apparent reduction in virulence in laboratory strains of mice *in vivo*. This may be due to the fact that the ROP5/17/18 complex in type I strains is so efficient in disarming the IRGs in mice that it masks the phenotype of the *ROP54* knockout in this context (10, 14, 26). Examination in wild-type strains of mice or other hosts that can resist type I parasites may expose virulence differences with the knockout of *ROP54* (46). In contrast, disruption of *ROP54* in type II parasites resulted in a 2-log decrease in virulence, even though growth in culture was unaffected (Fig. 4E and 5). Whereas the other ROP kinases and pseudokinases tend to be highly polymorphic across strains, the ROP54 amino acid sequences across type I, II, and III strains are nearly identical, with only 1 amino acid change. This suggests that this effector may play the same role in these diverse strains, although it is also possible that ROP54 expression levels may differ or that its activity may be altered by differences in its partners.

We showed that *∆rop54_II_* parasites are susceptible to the IFN-γ-mediated antimicrobial response *in vivo* and *in vitro*, suggesting that the *∆rop54_II_* parasites lack an immunosuppressive function (Fig. 6A to D). The susceptibility of the *∆rop54_II_* parasites correlated with the increased GBP2 loading on the vacuoles of *∆rop54_II_* parasites, while IRGb6 loading was sustained (Fig. 6E; see also Fig. S6 in the supplemental material). These data collectively suggest that the virulence defect observed in *∆rop54_II_* parasites *in vivo* is due to the GBP2 innate immune response (Fig. 5). GBPs play a significant role in controlling *Toxoplasma* infection, as IFN-γ-primed MEFs lacking GBP^chr3^ are deficient in parasite clearance (23). Multiple GBPs are likely to be important for host resistance, as complementation of GBP^chr3^-disrupted MEFs with *GBP2* was not sufficient to control parasite burden (23). However, GBP2^−/−^ mice exhibit an increased susceptibility to *Toxoplasma* infection *in vivo*, and GBP2^−/−^ MEFs are unable to limit parasite replication *in vitro* (25). Our data indicate that the pseudokinase ROP54 modulates immune loading of GBP2 (Fig. 6E), suggesting that it may represent a parasite strategy to evade the GBP2-mediated immune response. It is not known whether ROP54 functions in conjunction with an unidentified active ROP kinase to phosphorylate GBP2 (in a manner similar to the ROP5/ROP18 complex). It is also not known whether ROP54 may have potential roles in disarming other members of the IRG or GBP family, which will be the focus of future studies.

## MATERIALS AND METHODS

### Parasite and host cell culture.

*T. gondii* type I RH*∆ku80* and type II Pru*∆ku80* parental strains and the resulting modified strains were maintained in confluent monolayers of human foreskin fibroblast (HFF) host cells as previously described (47). Immortalized C57BL/6J macrophages were donated by Kenneth Bradley (UCLA). Bone marrow-derived macrophages (BMDMs) were donated by Steven Bensinger (UCLA).

### Antibodies used for Western blot assays and IFAs.

Hemagglutinin epitope tags were detected with mouse monoclonal antibody (MAb) HA.11 (Covance) and rabbit polyclonal antibody (pAb) anti-HA (Invitrogen). Flag epitope tags were detected with mouse anti-Flag MAb M2 (Sigma). Rabbit anti-ROP5 was received from David Sibley (Washington University, St. Louis, MO). Mouse MAb anti-ROP7, rat pAb anti-ROP9, and rabbit pAb anti-ROP13 antibodies were generated in the Bradley laboratory (33, 48). IRGb6 was detected with a goat pAb antibody (Santa Cruz Biotechnology). Rabbit anti-GBP2 pAb was received from Jorn Coers from Duke University (49). Mouse anti-SAG1 (DG52) MAb and rabbit anti-SAG1 pAb were both obtained from John Boothroyd at Stanford University (50).

### Endogenous tagging of TGME49_210370.

To endogenously tag TGME49_210370, the C terminus of the gene was PCR amplified with primers P1/P2 (primers are listed in Table S1 in the supplemental material) from PruΔ*ku80* and RHΔ*ku80* genomic DNA, T4 processed, and ligated using ligase-independent cloning (LIC) into 3×HA- or 2×Strep 3×Flag-tagging plasmids which contained the selectable marker *HXGPRT* as previously described (47). Fifty-microgram aliquots of the tagging constructs were linearized with PstI and transfected into PruΔ*ku80* and RHΔ*ku80* parasites. Stably transfected parasites were selected with MX medium (50 µg/ml mycophenolic acid and 50 µg/ml xanthine) and cloned using the limiting dilution method (51).

10.1128/mSphere.00045-16.1 Table S1 Primers used in this study (all primers are listed in the 5′-to-3′ direction; please refer to Materials and Methods in the main text for descriptions of the primers) Download Table S1, PDF file, 0.1 MB. Copyright © 2016 Kim et al. 2016 Kim et al. This content is distributed under the terms of the Creative Commons Attribution 4.0 International license.

10.1128/mSphere.00045-16.2 Table S2 Proteins identified via mass spectrometry of ROP54 immunoprecipitation (IP-Western blotting data demonstrated that ROP54 does not form complexes with ROP5; therefore, proteins that had a higher score than ROP5 that had either a predicted hypothetical protein, signal peptide, or cell expression profile similar to an ROP are listed) Download Table S2, PDF file, 0.04 MB. Copyright © 2016 Kim et al. 2016 Kim et al. This content is distributed under the terms of the Creative Commons Attribution 4.0 International license.

### IFA.

*T. gondii* strains were used to infect coverslips with a confluent monolayer of HFFs under the indicated time constraints for the IFA analyses. The coverslips were fixed in 3.7% formaldehyde–phosphate-buffered saline (PBS) for 15 min and then blocked and permeabilized in 3% bovine serum albumin (BSA)–0.2% Triton X-100–PBS for 30 min. The samples were then incubated with primary antibody diluted in 3% BSA–0.2% Triton X-100–PBS for 1 h at room temperature. The coverslips were then washed in PBS (5 times for 5 min each) and treated with secondary antibodies Alexa 488-conjugated goat anti-mouse and/or Alexa 594-conjugated goat anti-rabbit (Molecular Probes) diluted 1:2,000 in 3% BSA–0.2% Triton X-100–PBS (27, 52).

### Evacuole assay.

Evacuoles were assessed as previously described (5, 33). Extracellular ROP54HA_II_ parasites were treated with prechilled Dulbecco’s modified Eagle’s medium containing 1 µM cytochalasin D (Sigma). The parasites were then added to prechilled confluent monolayers of HFFs for 20 min. The coverslips were washed, and warm medium was added for 20 min. The coverslips were then washed with PBS and an IFA was performed as explained above.

### Disruption of *ROP54*.

To disrupt *ROP54*, the 5′ and 3′ regions flanking the *ROP54* gene were PCR amplified from PruΔ*ku80* and RHΔ*ku80* genomic DNA with primers P3/P4 and P5/P6 and ligated into the pMiniGFP.ht-DHFR knockout plasmid (48). Fifty-microgram amounts of the plasmid were linearized with *XbaI* and transfected into ROP54 HA-tagged parasite lines. The parasites were selected with 1 µM pyrimethamine, and knockouts were cloned via limiting dilution and identified by lack of HA staining in IFA and Western blot assays. The knockouts for type I and type II ROP54 were designated clones *∆rop54_I_* and *∆rop54_II_* (48).

### Complementation of *ROP54*.

The endogenous locus of *ROP54* was PCR amplified with primers P7 and P8 from genomic DNA from the ROP54HA_II_ strain. The PCR product contained the endogenous promoter, *ROP54* gene, 3×HA tag, and the *HXGPRT* 3′-untranslated region from the tagging construct. The amplicon was ligated into a complementation vector with the 3′ and 5′ flanks of the deleted *Ku80* locus and selectable marker *HXGPRT* (provided by Vern Carruthers, University of Michigan) (53). The plasmid was linearized with BssHII, transfected into the *Δrop54_II_* clone, and selected with MX medium. A ROP54 complement clone (ROP54c_II_) was generated using limiting dilution, and complementation was assessed by IFA and Western blot analysis (48).

### Macrophage clearance assay.

For macrophage clearance assays, RAW 267.4 cells were seeded at 1 million cells per T25 flask and activated with 100 units/ml of IFN-γ (Millipore) and 10 ng/ml of lipopolysaccharide (LPS; Sigma). The ROP54HA_II_, ∆*rop54_II_*, and ROP54c_II_ parasite strains were used to infect the RAW 267.4 cells at a multiplicity of infection (MOI) of 1 for 20 h, and the inoculum was confirmed via plaque assay. Total genomic DNA of each flask was isolated by using a DNA isolation kit (Promega). The amount of *Toxoplasma* and RAW 267.4 genomic DNA was quantified by qPCR (BioRad). *TgACT1* was amplified with primers P15 and P16, and BALB/c *actin* was amplified with primers P13 and P14, using 2× SYBR green stain (BioRad). The Δ*C_T_* values were calculated based on the amount of *TgACT1* relative to BALB/c *actin* (36–38). The *∆rop54_II_* and ROP54c_II_ values were then normalized to the value for ROP54HA_II_ to determine DNA amounts of the strains relative to that in the parental parasite strain.

### *In vitro* viability assay.

The *in vitro* viability assays, BMDMs were seeded at 1 million cells per T25 flask and activated as described above. The ROP54HA_II_, ∆*rop54_II_*, and ROP54c_II_ parasite strains were used to infect the BMDMs at an MOI of 1 for 20 h. The inoculum was confirmed via plaque assay. Parasites were mechanically disrupted with syringe lysis via a 17-gauge needle syringe and used to infect HFF monolayers with serial dilutions. Plaques were enumerated at 10 days postinfection, and the average number of live parasites per milliliter was calculated. Averages of *∆rop54_II_* and ROP54c_II_ parasite plaques were then normalized to the ROP54HA_II_ values to determine the relative fold changes in plaques per milliliter between the parasite strains (38).

### Plaque assays.

HFF monolayers were seeded onto 24-well plates and allowed to grow to confluence for plaque assays. These host cells were infected with an inoculum of each parasite strain, and plaques were allowed to grow for 6 days for type I parasites and 10 days for type II parasites (54). Each well was fixed with ice-cold methanol for 5 min, and the areas of the individual plaques were measured using the Zen imaging program (Zeiss).

### Western blot assay.

Extracellular parasites were lysed in Laemmli sample buffer (50 mM Tris-HCl [pH 6.8], 10% glycerol, 2% SDS, 1% 2-mercaptoethanol, 0.1% bromophenol blue) and heated at 95°C for 5 min in preparation for the Western blot assays. Samples were then separated by SDS-PAGE and transferred to nitrocellulose membranes (Maine Manufacturing, LLC). Equivalent loading of protein in each well was confirmed by counting parasites and verified by staining with antibodies against a loading control protein (52).

### Light microscopy and image processing.

IFA and plaques assay results were visualized on an Axio Imager.Z1 fluorescence microscope (Zeiss) as previously described (55). Images were collected using the AxioCam MRm charge-coupled-device camera and Zeiss Zen imaging software. Image stacks were collected at *z*-increments by using the “optimal slice” tool of the imaging software. The highest-quality images from the stack were deconvolved by using a point-spread function to generate a maximum intensity projection (MIP) (52).

### Semipermeabilization of host cell membranes for detection of ROPs on PVM.

To detect ROPs on PVM via semipermeabilization, confluent monolayers of HFFs were seeded onto coverslips and infected with ROP54HA_II_ parasites at the indicated time points. The samples were washed quickly with PBS and fixed in 4% formaldehyde (Polysciences) for 10 min at room temperature. The fixed coverslips were quenched with 100 mM glycine–PBS for 5 min at room temperature. The cells were permeabilized with either 0.002% digitonin–PBS (made fresh for each experiment) for 2.5 min at 4°C or 0.01% saponin–PBS for 30 min at room temperature. The samples were placed in blocking buffer (10% fetal calf serum [FCS]–PBS) for 30 min at room temperature to prevent nonspecific binding of the antibodies. Primary antibodies were diluted in blocking buffer (1:300 for MAb HA.11 [Covance], 1:300 for pAb ROP5 [Sibley], 1:100,000 for mouse SAG1 [DG52], and 1:100,000 for rabbit pAb SAG1) and used to probe the coverslips at room temperature for 1 h. The secondary antibodies Alexa 488-conjugated goat anti-mouse and Alexa 594-conjugated goat anti-rabbit (Invitrogen) were diluted at 1:2,000 in blocking buffer and added to the samples for incubation for 1 h (27). The coverslips were mounted in Vectashield (Vector Labs.) or ProLong Gold (Molecular Probes) and viewed with an Axio Imager.Z1 fluorescence microscope (Zeiss).

### *In vivo* virulence assays.

C57BJ/B6 mice (Jackson Laboratory) were injected i.p. with ROP54HA_II_, *∆rop54_II_*, or ROP54c_II_ parasites at doses of 500, 5,000, and 50,000 parasites (*n* = 4 mice/dose) (14). IFN-γR^−/−^ mice were acquired from Jane Deng laboratory (UCLA) and i.p. injected with 5,000 parasites. Parasite viability from the injections was verified by plaque assay immediately after infecting the mice. Mice were carefully monitored for 21 days to observe for weight loss and in accordance with institutional guidelines approved by the UCLA Animal Research committee.

### *In vivo* competition assay.

A mixed aliquot of ~60% *∆rop54_II_* and ~40% ROP54HA_II_ was made at a dose of 50,000 parasites. The mixed dose was i.p. injected into C57BJ/B6 mice, and the ratio of the mixed inoculum was confirmed by IFA. On days 4 and 7, the mice were sacrificed and peritoneal lavage samples were collected with wash buffer (1% FCS–5 mM EDTA in PBS). The cells collected from the lavage fluid were mechanically disrupted to liberate parasites. Confluent HFFs were infected with the parasites for 40 h. The coverslips were fixed and stained for IFA, and the ratios of ROP54HA_II_ and *∆rop54_II_* parasite vacuoles were determined. Spleens were also harvested on day 7 and homogenized in 1 ml of PBS. The homogenate was mechanically disrupted with sequential passage through 18-, 25-, and 27.5-gauge needles and used to infect a confluent monolayer of HFFs for 40 h. The monolayer was examined by IFA, and the numbers of ROP54HA_II_ and *∆rop54_II_* parasite vacuoles were determined.

### Immunoprecipitation.

For the immunoprecipitation assays, extracellular ROP54SF_II_ parasites were harvested and lysed in 0.5% NP-40, 150 mM NaCl, and 1× protease inhibitor cocktail (Roche) on ice for 30 min. The lysate was centrifuged at 14,000 × *g* at 4°C for 20 min. The supernatant was incubated with streptactin beads (Iba) for 4 h at room temperature. The beads were washed and eluted with 10 mM desthiobiotin in lysis buffer (56). Ten percent of the eluate was used for Western blot analysis, and the remainder was analyzed by mass spectrometry.

### Statistical analysis.

All experiments with three or more independent experiments were analyzed using one-way analysis of variance (ANOVA) and the Student-Newman-Keuls method for pairwise analyses.
