# Supplementary figures and images for: The Rhoptry Pseudokinase ROP54 Modulates Toxoplasma gondii Virulence and Host GBP2 Loading
Source: mSphere. 2016 Mar 23;1(2):e00045-16. doi: 10.1128/mSphere.00045-16 (PMC4863586; doi:10.1128/mSphere.00045-16)

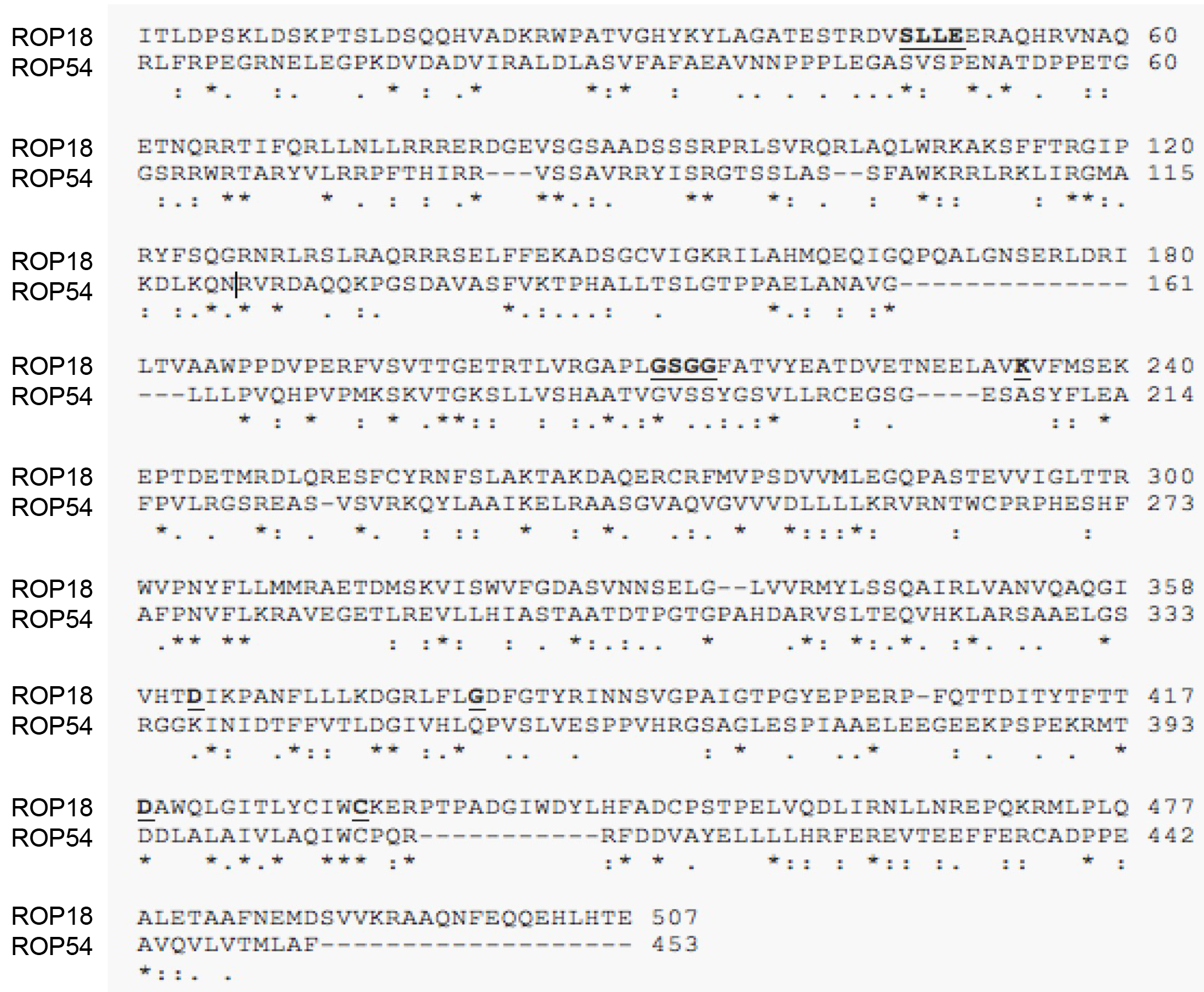

Supplement: Figure S1 [file sph002162044sf3.tif]

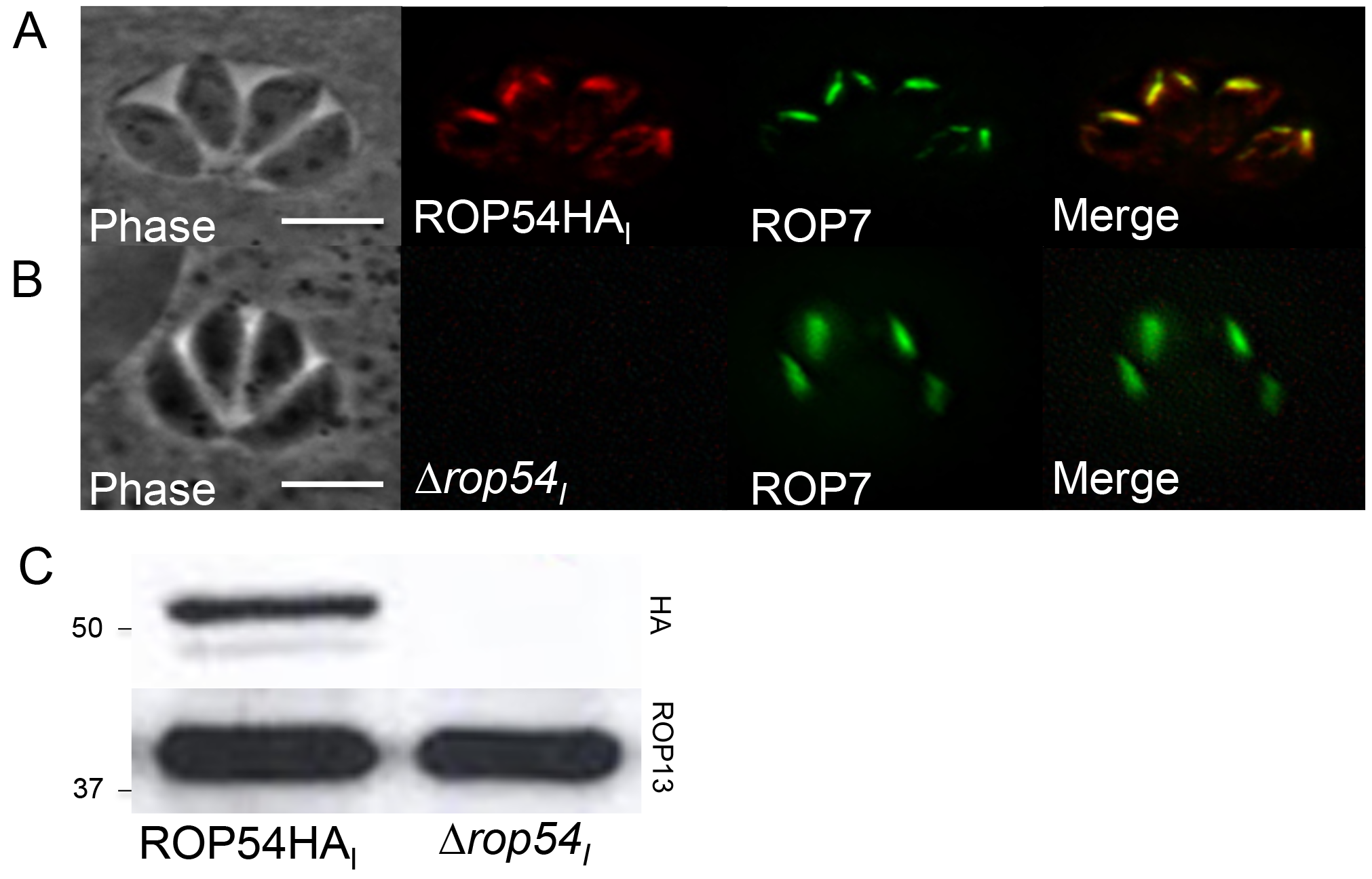

Supplement: Figure S2 [file sph002162044sf4.tif]

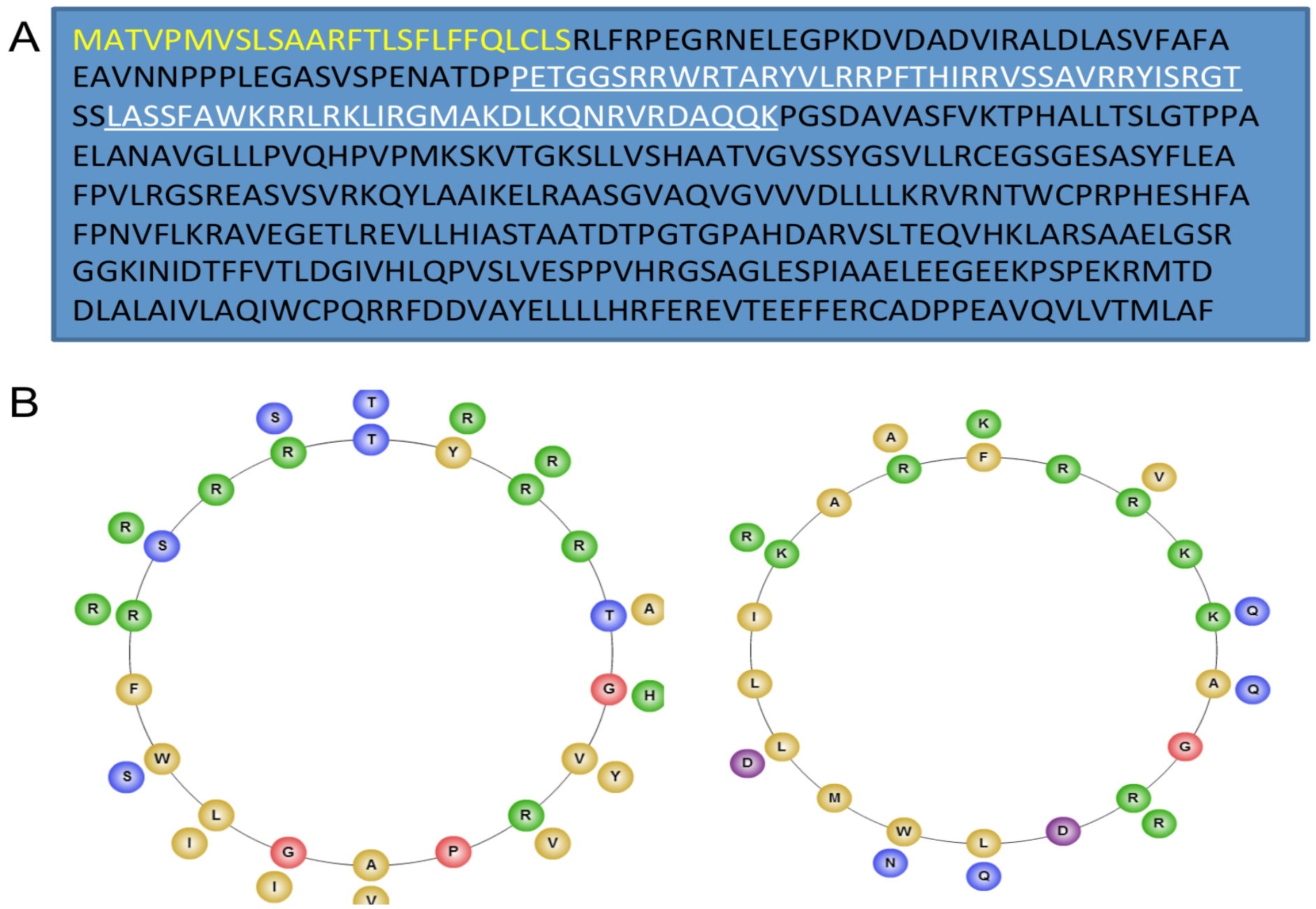

Supplement: Figure S3 [file sph002162044sf5.tif]

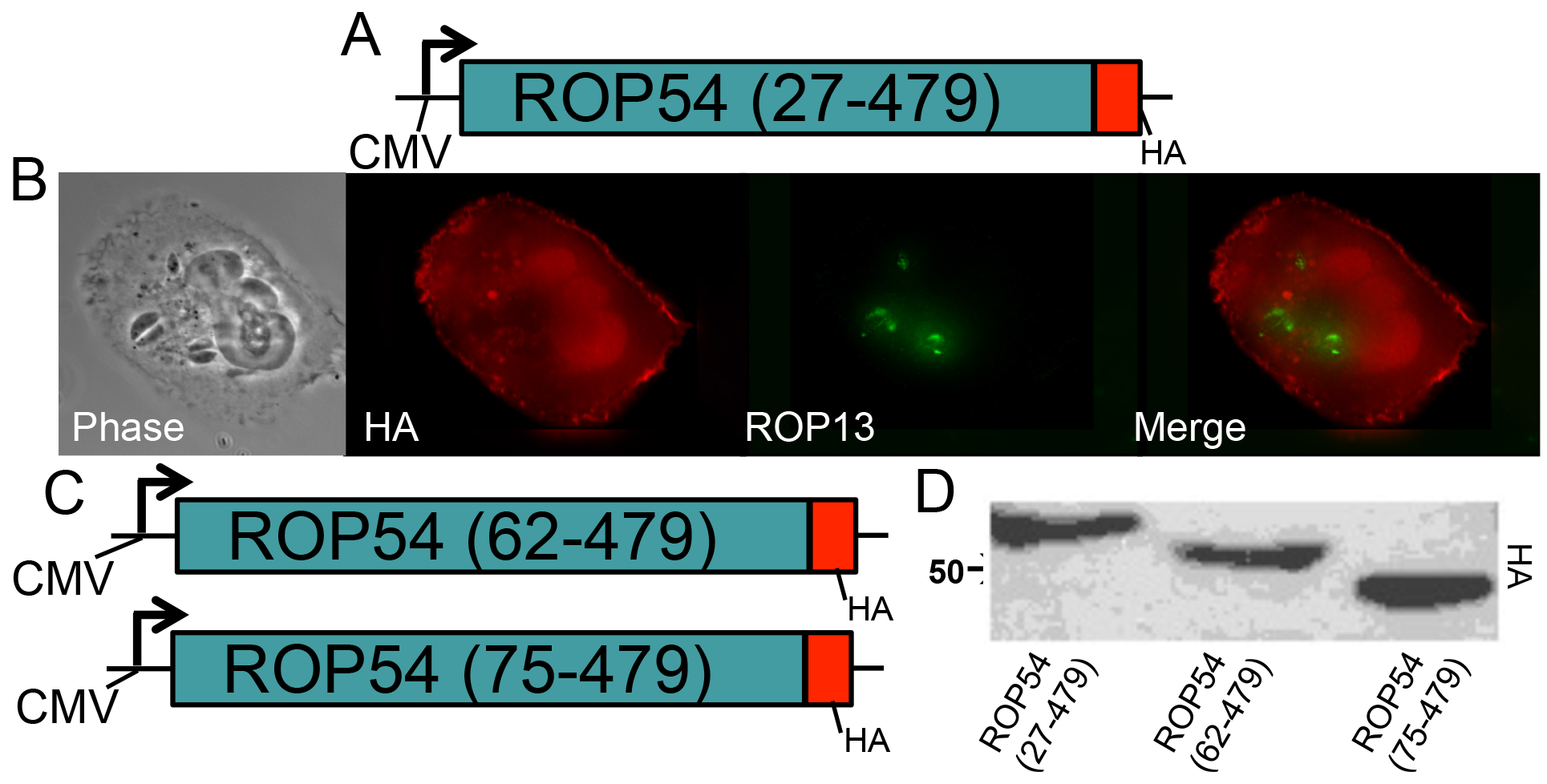

Supplement: Figure S4 [file sph002162044sf6.tif]

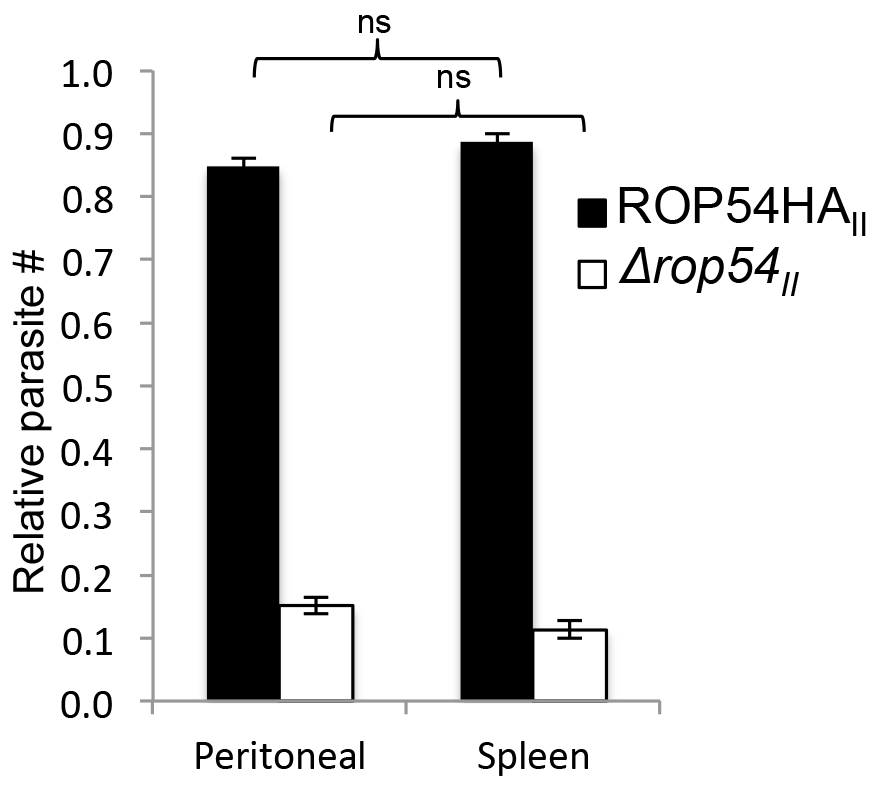

Supplement: Figure S5 [file sph002162044sf7.tif]

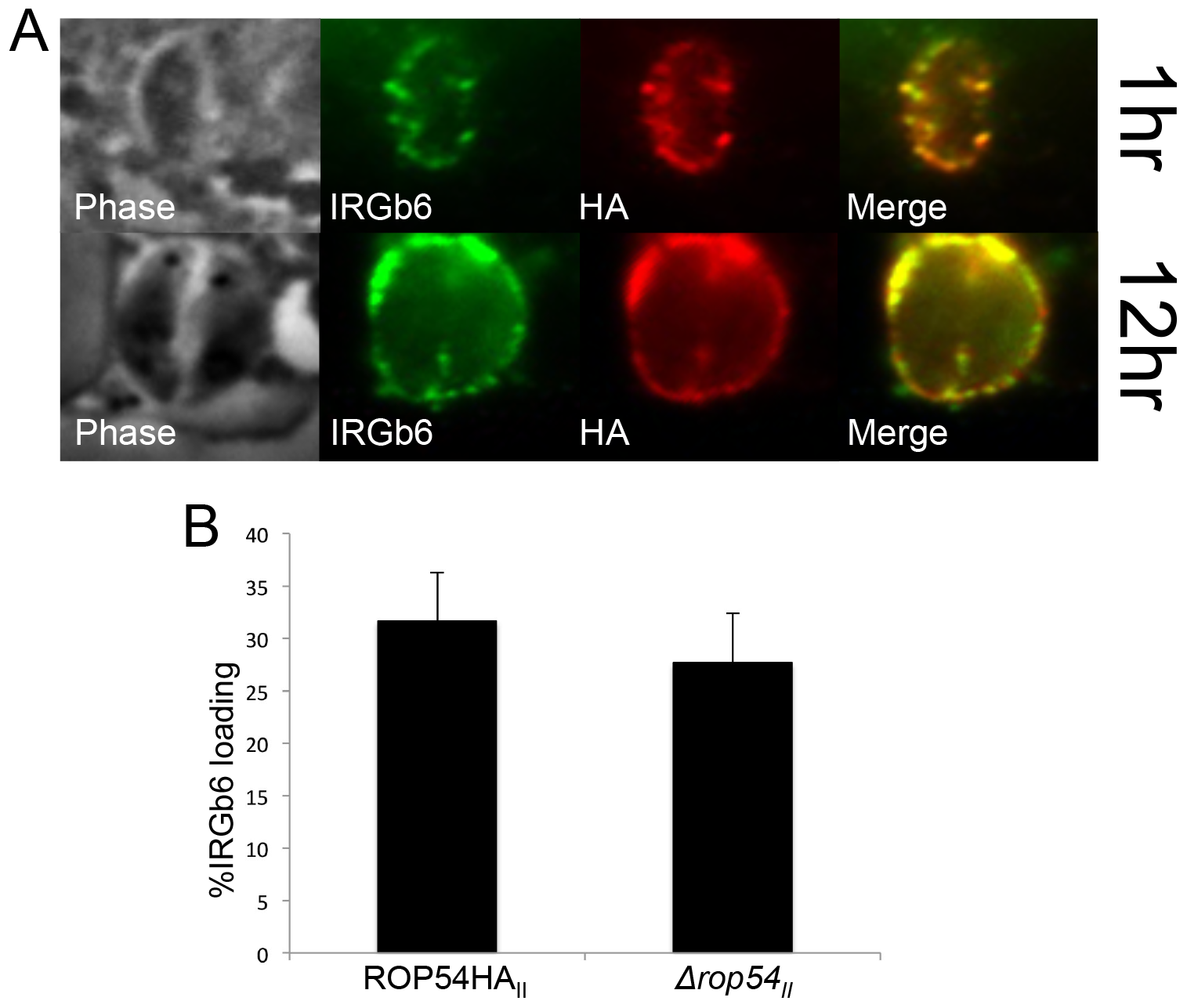

Supplement: Figure S6 [file sph002162044sf8.tif]
