# Supplementary material for: The Rhoptry Pseudokinase ROP54 Modulates Toxoplasma gondii Virulence and Host GBP2 Loading
Source: mSphere. 2016 Mar 23;1(2):e00045-16. doi: 10.1128/mSphere.00045-16 (PMC4863586; doi:10.1128/mSphere.00045-16)
Supplement: Table S1 [file sph002162044st1.pdf]

Name	Primers used in this study
P1	TACTTCCAATCCAATTTAGCCACTCGAGGGAGCCTCT
P2	TCCTCCACTTCCAATTTTAGCGAAAGCCAGCATTGTGACCAGG
P3	GTGTCGCGCTAGGTTTTAC
P4	GATCACTAGTCAAGTTGTATCTGGTTTCCC
P5	GATCAAGCTTCTCGGCAGAAAAGGACTGTG
P6	GATCGGGCCCGAGTCCCGGTTTTGCCTG
P7	TCTAGAGCTAGGTTTTACAAAGAGTCGG
P8	TCTAGAATCGATACCGTCGACCTC
P9	GATCCCATGGGCCGGCTGTTCCGGCCCG
P10	GATCGCGGCCGCGAAAGCCAGCATTGTGACCA
P11	GATCGATATCGCCACCATGGCGGTCAACAACCCACCG
P12	GATCGATATCGCCACCATGACGGGCGGTTCTCGTCGT
P13	GCTCCTAGCACCATGAAGATCA
P14	CAAGATGGTGAATGGTGAGCTC
P15	TCCCGTCTATCGTCGGAAG
P16	CCATTCCGACCATGATACCC
