# Supplementary material for: The Rhoptry Pseudokinase ROP54 Modulates Toxoplasma gondii Virulence and Host GBP2 Loading
Source: mSphere. 2016 Mar 23;1(2):e00045-16. doi: 10.1128/mSphere.00045-16 (PMC4863586; doi:10.1128/mSphere.00045-16)
Supplement: Table S2 [file sph002162044st2.pdf]

Gene Number	Protein type	Signal peptide	How similar to ROP cell expression	Score
**TGGT1_293690**	Profilin PRF	no	weak	500.697
**TGGT1_214790**	Gycoprotein cycles	no	weak	346.065
**TGGT1_306670**	Hypothetical protein	no	weak	272.045
**TGGT1_243910**	Cof family hydrolase subfamily protein	no	cycles	218.218
**TGGT1_221510**	Hypothetical protein	yes	yes	205.317
**TGGT1_237180**	Hypothetical protein	yes	yes	192.032
**TGGT1_312630**	Putative anonymous antigen-1	no	no	168.96
**TGGT1_252360**	ROP24 (incomplete catalytic triad)	yes	yes	152.549
**TGGT1_229210**	Putative small nuclear ribonucleoprotein polypeptide A'	no	no	128.525
**TGGT1_299210**	Product: CTP synthase	no	weak	125.174
**TGGT1_243950**	Putative prohibitin	yes	yes	120.463
**TGGT1_232340**	Protein phosphatase 2C domain-containing protein	no	no data	119.362
**TGGT1_222948**	Hypothetical protein	no data	no data	111.418
**TGGT1_232130**	Hypothetical protein	no	yes	107.386
**TGGT1_263090**	14-3-3 protein	no	weak	101.069
**TGGT1_306030**	Glutathione s-transferase, n-terminal domain containing protein	no	yes	98.034
**TGGT1_225550**	Phosphatidylserine decarboxylase	yes	no	96.871
**TGGT1_269190**	Glyceraldehyde-3-phosphate dehydrogenase GAPDH2	yes	yes	96.394
**TGGT1_411430**	Rhoptry protein ROP5	yes	yes	89.195
